# Tibia plateau fracture mapping and its influence on fracture fixation

**DOI:** 10.1186/s10195-019-0519-1

**Published:** 2019-02-26

**Authors:** Lorcan McGonagle, Tim Cordier, Bjorn C. Link, Mark S. Rickman, Lucian B. Solomon

**Affiliations:** 1 Orthopaedic and Trauma Service, Royal Adelaide Hospital, Port Road, Adelaide, SA 5000 Australia; 2 Department of Orthopaedic and Trauma Surgery, Lucerne Cantonal Hospital, Lucerne, Switzerland; 3 Centre for Orthopaedic and Trauma Research, The University of Adelaide, Adelaide, SA 5005 Australia

**Keywords:** Tibia, Plateau, Fracture, Fixation

## Abstract

**Background:**

Tibial plateau fracture classifications are based on anteroposterior radiographs. Precontoured locking plates are commonly used to treat such fractures. The aims of this study are to: (1) describe tibial plateau fracture anatomy in the axial plane and (2) assess whether current plating systems allow screws to be placed suitably.

**Materials and methods:**

A graphical tibial plateau template was developed. One hundred twenty-five tibial plateau fractures (four bilateral) were reviewed (80 men, 41 women; average age 45.5 years, range 21–77.7 years). The axial computed tomography (CT) slice 0.3–0.5 mm below the medial articular surface was reviewed in all cases. Fracture lines were drawn on the template. Four lateral locking plates were placed against a cadaveric adult tibia. Based on the projected screw directions, suitable fracture patterns were identified. Fractures were considered “suitable” if the screws passed 90 ± 22° to the fracture line.

**Results:**

Two hundred sixty-one different fracture lines were identified. One hundred thirty-four fractures involved the lateral plateau; 96 were suitable for lateral plating. Ninety fractures involved the medial plateau, 82 were treatable using the various plate positions on medial-posterior aspect of the medial plateau. Thirty-seven fractures were bicondylar; 20 were treatable with a posteromedial plate.

**Conclusions:**

Tibial plateau fractures follow consistent patterns, with most lateral and medial plateau fracture lines being in the sagittal plane, although there is greater variation medially. Positioning of modern locking plates will deal effectively with 72 % of all lateral plateau fractures and 91 % of medial plateau fractures.

**Level of evidence:**

Level 3.

## Introduction

Tibia plateau fractures are common injuries that often require surgical treatment. Most cases occur around the fifth to sixth decades, with men and women affected equally [1, 2]. Challenges with such fractures include gaining anatomical reduction of the articular surface and satisfactory internal fixation, within the constraints of local soft tissues and blood supply. The Schatzker and AO classification systems, commonly used for tibial plateau fractures, are based on anteroposterior radiographs of the knee (i.e., coronal plane) without consideration of fracture anatomy in the axial or sagittal planes [1, 3]. Understanding fracture anatomy is integral to choosing the correct surgical approach for fixation and the correct placement of plate and screws.

Locking plates are widely used for treatment of periarticular fractures, as they provide angular stability and may allow earlier motion and weight bearing, particularly when compared with fracture fixation with nonlocking plates [4, 5]. There has been renewed interest recently in the morphology of tibial plateau fractures, using computed tomography (CT) imaging [6, 7]. However, little is known about whether modern precontoured plates can be satisfactorily placed to deal with such fractures.

The primary aim of this study is to describe the anatomy of tibial plateau fractures in the axial plane. The secondary aim is to determine whether current plating systems are appropriate to deal with these fracture patterns. Appropriate fixation was defined based on biomechanical studies that suggested screws should not be positioned more than 22° from a perpendicular to the fracture line and that locking screws have the best interference strength and stiffness when inserted perpendicular to the plate [8, 9].

## Materials and methods

### Developing a template

CT scans of 50 knees with no bony pathology were identified from the radiographic database of our institution.

The axial slice for each tibial plateau 0.3–0.5 mm below the joint surface was identified. The maximum anteroposterior (AP) and mediolateral (ML) dimensions of all plateaus were measured. The average AP:ML ratio was 1:1.3, forming the basis of our template. A best-fit line was then drawn based on all the images to reproduce the curved outline of the tibial plateau. All images were viewed as a left tibia. In cases were the right tibia was scanned, the image was flipped horizontally electronically to resemble a left tibia.

This provided us with a graphical template upon which we drew our fractures. Fracture lines were plotted using *X*–*Y* coordinates for the entry and exit points on the cortical surface using a Microsoft Excel 97–2003 worksheet, as described previously by Mandziak [10].

### Patients

The inclusion criteria were tibia plateau fractures treated surgically between 2006 and 2014 in patients aged over 18 years, with a fine-cut preoperative CT scan of the injured knee at 0.3–0.5 mm.

Exclusion criteria were fractures treated nonoperatively, isolated tibial tubercle avulsion fractures, isolated anterior cruciate ligament (ACL)/posterior cruciate ligament (PCL) avulsion fractures, patients under 18 years, and in situ metalwork.

One hundred twenty-five tibial plateau fractures (four bilateral) fit the inclusion and exclusion criteria and were included in this study. There were 80 men and 41 women. The average age was 45.5 years (range 21–77.7 years). The mechanism of injury is outlined in Table 1.

**Table 1:** Mechanism of injury

Mode of injury	Number of cases
Motorbike accident	42
Pedestrian/cyclist versus car	20
Fall from height	19
Fall from standing height	16
Sporting accident	16
Crush injury	12

### Image analysis

All patients had a CT scan prior to definitive fixation. The axial CT images were reviewed. Fracture anatomy was drawn out using the tibial plateau grid described above. Emulating the technique used by Cole to configure tibial plafond fractures, we utilized the axial slice 0.3–0.5 mm below the medial articular surface [11]. Only two patients had a spanning external fixator at the time of CT. Fracture lines were drawn as “virtually” reduced fragments as per Molenaars [7], thereby correcting for any fracture displacement, including depressed fractures.

All fracture lines were drawn separately by two of the authors. A subsequent review of all images was carried out by two different authors to ensure agreement and consistency of fracture mapping. This included confirming the coordinates for all fracture lines. Analysis of more distal axial slices as well as reviewing the sagittal and coronal cuts was carried out to confirm the medial/lateral/bicondylar classification of fractures. Disagreement was resolved by consensus.

Postoperative radiographs/CT scans were not reviewed as part of this study.

### Cadaveric plate application

Two dried cadaveric adult tibiae (one left, right) with no soft tissue attachments were obtained from the University of Adelaide anatomy department. A selection of modern locking lateral locking plates were obtained:

- Periarticular proximal tibial locking plate (Zimmer, Warsaw, USA)
- NCB^®^ proximal tibia system (Zimmer, Warsaw, USA)
- 3.5-mm LCP proximal tibia plates (Depuy Synthes, West Chester, USA)
- Tibia PERI-LOC plate (Smith and Nephew, Memphis, USA)

**Fig. 1:**
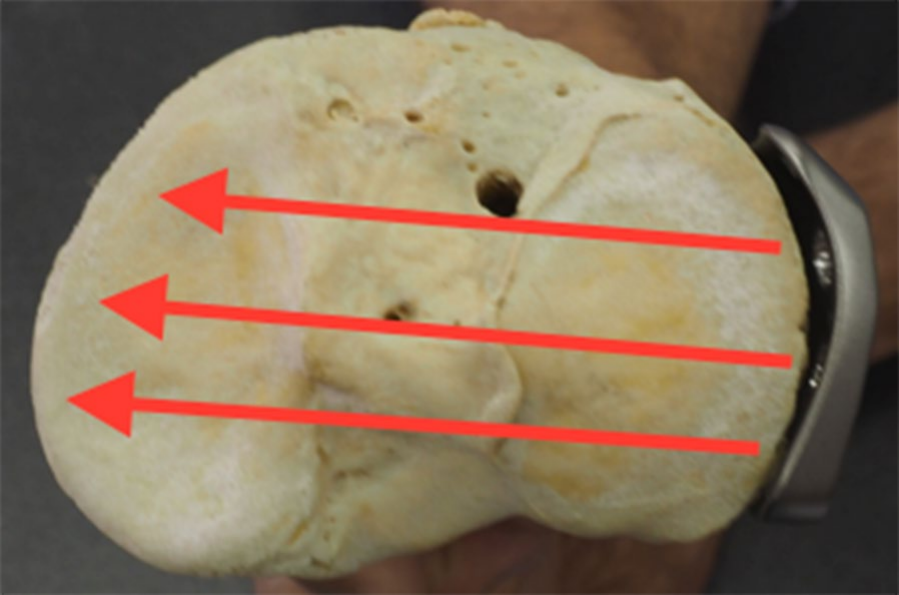
Apical view of locking plate placed against tibia (Zimmer NCB proximal tibia system)

**Fig. 2:**
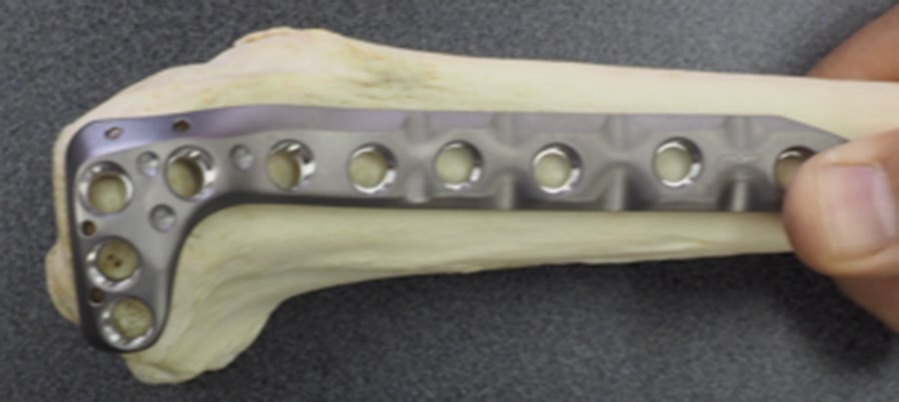
Lateral view of locking plate placed against cadaveric tibia (Zimmer NCB proximal tibia system)

These lateral locking plates were then placed against the cadaveric tibiae as per the respective manufacturer’s technique guide, as shown in Figs. 1, 2. Based on these images, we projected the screw directions as shown by the red arrows in Fig. 1. We then determined which fracture patterns were suitable for treatment with a lateral locking plate. Fractures were deemed suitable for fixation with a lateral plate if the screws would pass 90 ± 22° [8].

**Fig. 3:**
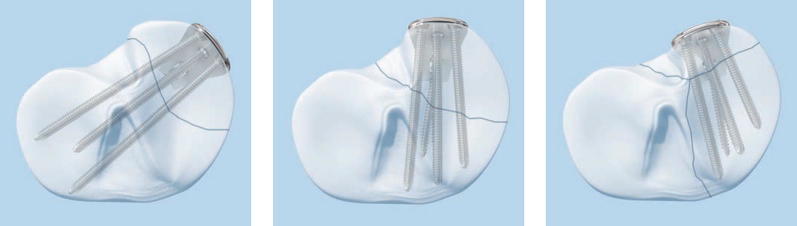
Potential medial plateau plate positions (3.5-mm LCP posteromedial proximal tibia plate, Synthes technique guide)

Plates on the lateral side are limited in their positioning by the anatomical shape of the plates, whereas on the medial side it is more the surgical approach and soft tissues that limit plate position. As a result of this, the plate position is potentially much more variable medially than on the lateral side, as shown in Fig. 3. Considering the range of possible medial plate positions from direct medial to posteromedial, we used the plates below for analysis.

- PERI-LOC VL 3.5-mm posteromedial proximal tibia locking plate (Smith and Nephew, Memphis, USA)
- 3.5-mm LCP posteromedial proximal tibia plate (Depuy Synthes, West Chester, USA)

Variation in possible screw trajectory was taken into account where appropriate.

## Results

### All fractures

**Fig. 4:**
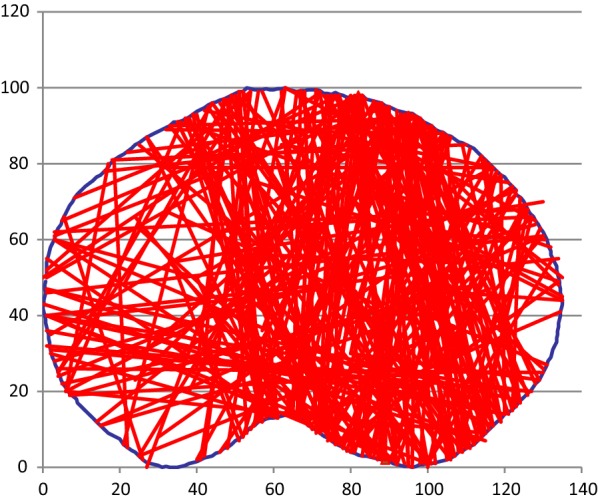
Superimposition of all fracture lines onto one template

In the 125 patients included in the study, 261 different primary fracture lines were identified, as depicted in Fig. 4.

### Lateral plateau fractures

**Fig. 5:**
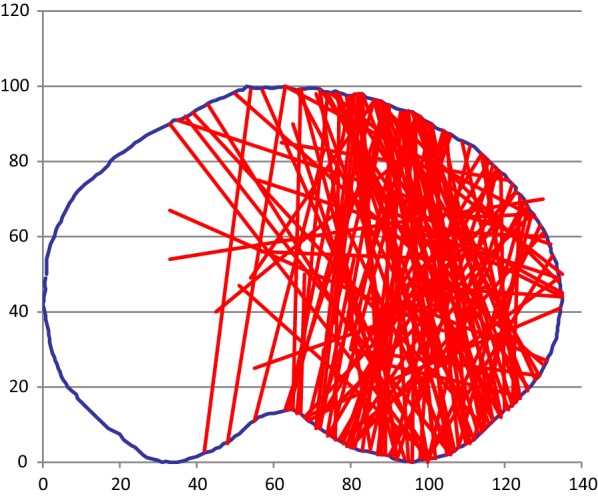
Lateral plateau fractures

One hundred thirty-four fractures involved the lateral plateau (Fig. 5). Eighty-four of these fractures were suitable for lateral plating.

### Medial plateau fractures

**Fig. 6:**
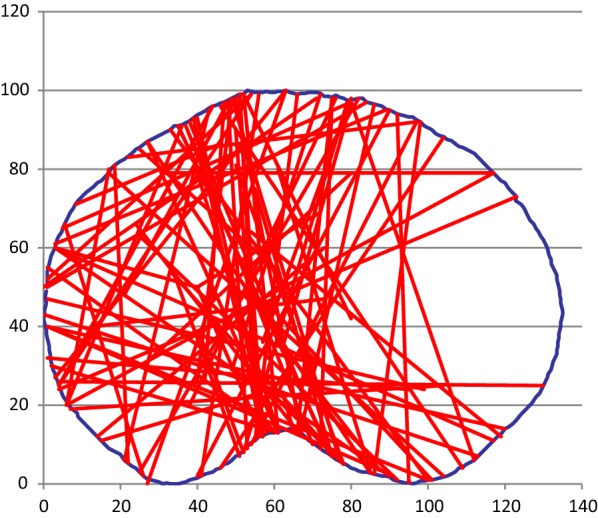
Medial plateau fractures

Ninety fractures involved the medial plateau (Fig. 6). Sixty-three of these fractures were treatable using the various plate positions on medial-posterior aspect of the medial plateau.

### Bicondylar fractures

**Fig. 7:**
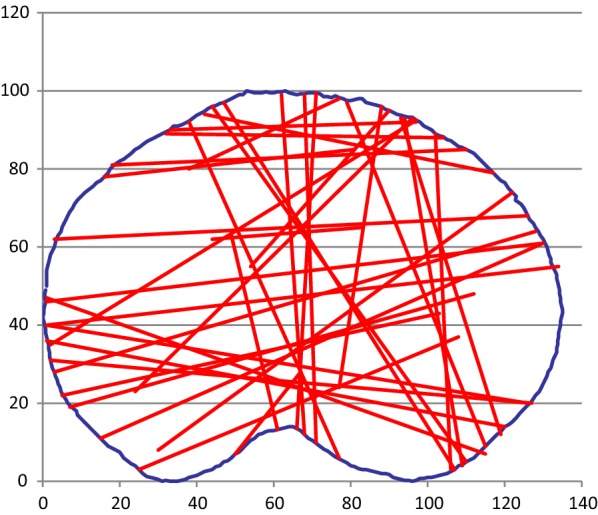
Bicondylar fractures

Thirty-seven fractures were bicondylar (Fig. 7). Such fractures are obviously bicondylar when seen in the coronal plane, whilst the sagittal plane fractures were deemed bicondylar as there was marked metaphyseal comminution (i.e., Schatzker 6 classification) upon reviewing the other CT slices. This comminution prevented medial/lateral classification. Twelve of these fractures were treatable with a posteromedial plate.

## Discussion

This study characterizes the axial anatomy of tibia plateau fractures, and shows the applicability of current plating systems. The results for our primary aim show that the most common fracture pattern was a sagittal plane fracture of the lateral plateau, being responsible for just under half of all tibial plateau fractures. Although sagittal plane fracture was also the most common type of fracture involving the medial plateau, the medial side exhibited much greater variation in fracture line orientation.

In a similar CT-based study, Molenaars [7] noted that a lateral split fragment was identified in 75 % of fractures, whilst our study identified that lateral plateau fractures accounted for just over half of all fractures. They also stated that 43 % of their fractures involved a posteromedial fragment, extended from the (antero)medial to the (postero)lateral side of the plateau. Our dataset shows that only 10 % of all fractures and 28 % of medial plateau fractures follow this (antero)medial-(postero) lateral pattern. Patient and mechanism of injury variables between these cohorts could be responsible for these differences.

Luo [6] used axial CT fracture anatomy to describe a “3 column fracture” concept (anterior, medial, posterior). This concept partially matched findings in our bicondylar fractures, although only 12/37 of our bicondylar fractures had a “posterior column” fragment, suggesting that this concept is not universally applicable.

Barei [12] described a posteromedial tibia fragment relative to the posterior femoral condylar axis (PFCA), showing that 74 % of bicondylar fractures include a posteromedial fragment. A similar study by Higgins [13] reported an incidence of 59 % of posteromedial fragments in bicondylar tibia plateau fractures. These two studies described a wide variation in the plane of the posteromedial fracture fragment as described by the major articular fragment angle, which varied from − 87 to + 52° and − 41 to + 23° relative to the PFCA, respectively. This wide variation in the plane of the fracture makes it difficult to predict plate/screw placement consistently.

Thirty-seven patients in our study had bicondylar injuries (including separate medial and lateral plateau fracture lines), although only 18 had a “posteriorly based fracture line of the medial plateau with the fracture line exiting the medial cortex” [13]. Why our bicondylar injury cohort has a lower proportion of posteromedial fragments is unclear, although different definitions of the fragment may be partly responsible.

The results for our secondary aim show that “correct” positioning of lateral locking plates will result in satisfactory positioning of locking screws relative to the fracture line in 71 % of all lateral plateau fractures and 91 % of all medial plateau fractures.

Medial plateau fractures in numerous different planes are treatable as there is wide variation in the potential positioning of the medial locking plates. Lateral locking plate position is relatively fixed. This means that a much narrower range of fracture planes is treatable with these plates.

To the best of our knowledge, no previous study has assessed tibial plateau fracture orientation and plate/screw positioning to the same degree as in this study. However, the importance of placing plates as parallel as possible to fracture lines has been demonstrated by Weaver [14]. Their series on condylar fractures showed that lateral plating alone was effective in maintaining reduction in medial fracture lines in the sagittal plane, but not medial fracture lines in the coronal plane. Those coronal plane medial fractures treated with dual plating had significantly less loss of reduction than isolated lateral plating. These findings are supported by Gosling 2005, who noted that a single lateral Less Invasive Stabilization System (LISS) plate was associated with a secondary loss of reduction in 14 % of complete articular fractures, although crucially this study excluded cases which also had a medial plate [15].

The importance of isolated lateral locking plate versus dual plating has been assessed biomechanically with some variations in results, suggesting that dual plating may not be that important. However, none of these fracture models that used a medial plate had screws that crossed a medial articular fracture at 90°, nor did any of them recreate the posteromedial fracture in the coronal plane [16, 17]. However, Zeng [18] showed that cortical screws via a posterior T-shaped buttress plate allowed the least subsidence of the posteromedial fragment when compared with anteroposterior lag screws, an anteromedial limited contact dynamic compression plate (LC-DCP), a lateral locking plate.

This study has a number of limitations. Firstly, all fractures are drawn as straight lines based only on their entry and exit coordinates. In reality, many were curved or “S” shaped. Secondly, only fracture patterns in the axial plane were considered. Fracture patterns in the sagittal and coronal planes also need to be taken into account when making a preoperative plan. Thirdly, we considered locking screws passing at 90 ± 22° to the fracture line as acceptable [8]. Further research on how much the locking screw angle across fracture fragments affects stability is needed.

Note that not every fracture line needs to be treated with a separate plate/screw construct. Fracture lines that are parallel or nearly parallel can be managed with the same plate/screw construct.

In conclusion, tibial plateau fractures follow consistent patterns, with most lateral and medial plateau fracture lines being in the sagittal plane, although there is greater variation medially.

Positioning of modern locking plates will deal effectively with 72 % of all lateral plateau fractures and 91 % of medial plateau fractures.

Careful analysis of preoperative CT scans, especially in the presence of medial fractures, is required in order to formulate an appropriate surgical plan using currently available locking plate technology.
